# Affordances of Multidisciplinary Team Meetings as Team-Learning Activity: A Constraints-Led Approach for Continuous Health Profession Education

**DOI:** 10.5334/pme.1733

**Published:** 2025-11-13

**Authors:** Lisa-Maria van Klaveren, Patric C. Nordbeck, Wendy Homan, Saskia Peerdeman, Stéphanie van der Burgt

**Affiliations:** 1 Institute of Education and Training, Amsterdam UMC location University of Amsterdam, Amsterdam, The Netherlands; 2 Amsterdam Public Health (APH), Quality of Care & Personalized Medicine, Amsterdam, The Netherlands; 3 University College Groningen, University of Groningen, Groningen, The Netherlands; 4 Faculty of Psychology, Lund University, Lund, Sweden; 5 Teaching and Learning Center, Faculty of Medicine, Amsterdam UMC location University of Amsterdam, The Netherlands; 6 Faculty of Medicine, Amsterdam UMC location Free University Amsterdam, Amsterdam, The Netherlands; 7 Amsterdam Public Health (APH), Quality of Care, Amsterdam, The Netherlands

## Abstract

The complexity of modern healthcare continues to evolve, emphasizing the need for comprehensive collaboration among professionals from diverse backgrounds, patients, and their families to integrate care. Multidisciplinary team meetings (MDTMs) have emerged as an opportunity to advance interprofessional collaborative practice. However, despite their growing prevalence in clinical and educational contexts, a systematic understanding of their potential as team-learning activity remains limited. To optimize their transformative potential across both contexts, it is critical to identify factors that support team learning and to explore how MDTMs can promote interprofessional development at team and individual levels. This paper presents a conceptual framework for understanding team learning in MDTMs from an ecological-dynamical perspective and offers a constraints-led approach for the purposeful design and implementation of simulated MDTMs as team-learning activity in continuous health profession education. We analyze constraints at three interdependent system levels: 1) healthcare and faculty context, 2) work teams, and 3) team members, and examine how they shape opportunities for team learning. By selecting, designing, and varying relevant constraints, educators can enhance simulated MDTMs as team-learning activity, ensuring that learners share information, co-construct meaning, and engage in constructive conflict. This framework offers actionable principles for backward design, enabling educators to tailor simulated MDTMs to real-world complexity and variability. By presenting an ecological-dynamical framework and a constraints-led approach, this paper contributes to the discourse on interprofessional collaborative practice and education, leveraging the transformative potential of MDTMs to enhance integrated care, foster shared cognition, and support the development of interprofessional competence.

## Introduction

Healthcare systems are continuously changing to accommodate increasingly complex care needs. These shifts become apparent at three interdependent levels: First, the prevalence of patients experiencing multiple physical and/or mental long term conditions has surged, particularly within an aging population [1,2]. Addressing their care needs requires a comprehensive approach that extends beyond the scope of a single, increasingly specialized professional. Second, there is a noticeable empowerment of patients and their families, emphasizing their autonomy and active participation in decision-making [3]. This shift underscores the importance of collaborative care models that acknowledge and incorporate the preferences and values of the individual and their loved ones receiving and providing care. Third, the increasing economic struggle of modern healthcare systems leads to the urge to reduce the average length of hospital stays [4,5]. This increases the number of patient transitions between settings, encompassing inpatient and outpatient facilities, as well as rehabilitation centers and home care. The challenge lies in ensuring continuity of care across these settings, emphasizing the need for integrated care [6].

Consequently, integrating care has increasingly become a collaborative process that transcends the expertise of individual professionals and the confines of single settings [7]. Interprofessional collaborative practice offers a means to navigate this growing complexity, emphasizing the active and ongoing partnership among diverse professionals, patients, and their families to ensure high-quality and continuity care [8]. Schot et al. [9] identify three key components essential for effective collaboration. First, team members must bridge professional, social, physical, and task-related gaps to ensure seamless coordination. Second, they must negotiate overlapping and opposing expertise, roles and responsibilities, requiring interprofessional competence, understood as the integration of knowledge, skills, and attitudes that enables effective communication, role clarification, and conflict resolution [10,11,12]. Finally, collaboration must be supported by dedicated (virtual) spaces where interprofessional teams can engage in meaningful interactions to provide integrated care. In clinical practice, multidisciplinary team meetings (MDTMs) provide joint spaces where team members bridge gaps, negotiate and coordinate care. Besides improving decision-making efficiency and integration of care, MDTMs also serve as valuable contexts for interprofessional education in which students from two or more professions learn from, about and with each other through structured dialogue and guided reflection [13,14,15]. Observation and participation in real-world MDTMs during internships, clerkships, traineeships, and residency programs, as well as engagement in simulated MDTMs within curricula, aim to enhance interprofessional competence development [16,17,18].

### Multidisciplinary Team Meetings

Although MDTMs are increasingly implemented and studied across clinical and educational settings, clear definitions and a systematic understanding of their broader functions remain limited. For instance, the UK’s National Health Service (NHS) describes MDTMs as “a meeting of a group of professionals from one or more clinical disciplines who together make decisions regarding recommended treatment”, while the Dutch Federation of Medical Specialists (FMS) specifies the “minimum presence of three different specialists authorized to initiate the care process to systematically discuss and decide on diagnostic and treatment planning” [19,20]. Research further characterizes MDTMs as forums in which cases are presented, expertise is shared, coordinated and reviewed, and care management decisions are discussed and documented to ensure timely (final) diagnosis and treatment, evidence-based management, and continuity of care [21,22,23,24,25]. While these perspectives converge on the premise of MDTMs as team activities in which members collaborate to deliver high-quality and continuous care, they often overlook whether and how teams learn during these meetings. Team learning refers to recurrent patterns of behaviors in teams, such as sharing of information, ideas or opinions, co-constructing meaning, and engaging in constructive conflict, through which members build mutual understanding and agreement that circularly lead to change or improvement [26,27]. This gap matters because team learning has important implications across multiple levels: At the organizational level, it supports integrated care by uncovering possibilities across professions, disciplines, and settings attuned to individual care needs; at the team level, it fosters shared cognition through the dynamic alignment of perceptions and actions that sustain coordination and adaptability; and at the individual level, it enables professionals to develop interprofessional competence, providing the flexibility to act appropriately at the right time and place [27,28,29]. Yet without systematically conceptualizing MDTMs as team-learning activity, current education and practice risk underutilizing their transformative potential. Moreover, and given their inherently complex and dynamic nature, MDTMs pose challenges that conventional conceptual frameworks and instructional approaches may not fully address [30,31,32].

### Purpose of this Paper

Against this backdrop, the purpose of this paper is twofold: First, to provide a conceptual framework for systematically understanding team learning in MDTMs from an ecological-dynamical perspective. Second, to offer practical guidance, using a constraints-led approach, for optimizing their potential as team-learning activity. To that end, we begin by outlining team-learning behaviors (sharing, co-construction, constructive conflict) during MDTMs and intra- and inter-system processes (team reflexivity, boundary crossing) that support the development of shared cognition and interprofessional competence [28]. Building on this foundation, we examine the constraints that simultaneously limit and shape team learning, described at three interdependent system levels: 1) healthcare and faculty context, 2) work teams, and 3) team members. Particular attention is paid to work teams and task demands, as educators can deliberately select and vary them to stimulate team learning. Finally, we leverage a constraints-led approach to offer concrete principles for the design and implementation of simulated MDTMs as team-learning activity in continuous health profession education.

## Ecological-Dynamical Framework for MDTMs as Team-Learning Activity

Dynamic systems theory is increasingly applied to conceptualize teamwork and learning as well as the healthcare system more broadly, offering a lens to explain complex, non-linear behavior that emerges from interdependent components across system levels [28,30,31,32]. Complementing this, the ecological perspective focuses on the reciprocal relationship between individuals and their environment, introducing the concept of affordances to understand how behaviors emerge in a given situation, such as an MDTM and develop over time [33,34,35]. Affordances refer to the possibilities for action that exist in a given situation, reflecting the fit between what a person or team can do and the constraints imposed by the environment and task [34,36]. For example, a particular chair affords sitting for a human of a particular size, but not for an elephant, or if the chair is made of paper. Beyond sitting, the same chair may also afford standing on, leaning against, yelling at, or using as a barricade. This concept extends to Landscape of Affordances, which is an abstract space of all available action possibilities, where availability is shaped by what is physically, socially and culturally possible in the given situation [37].

### Team Learning

Team learning refers to behaviors that team members engage in during team activities and the mechanisms that circularly generate change. Behaviors are immediate actions of team members during an MDTM, while mechanisms describe longer-term processes through which teams collectively reflect, adapt, and develop. Together, these dynamics enhance care integration while fostering shared cognition at the team level and interprofessional competence at the individual level.

#### Team-learning behaviors

Decuyper and colleagues [28] identify three basic team-learning behaviors: sharing, co-construction and constructive conflict, that describe the possible actions during team-learning activities. *Sharing* involves exchanging information, knowledge, and feedback among team members [27,28,29]. It allows for the identification of diverse standpoints and helps negotiate roles, laying the foundation for learning [35]. *Co-construction* refers to the collaborative development of new understandings, ideas or solutions by integrating and synthesizing contributions from different domains [28]. This process involves active dialogue and coordination among team members to build mutual understanding and meaning [26]. By combining unique standpoints and expertise, teams can create innovative and comprehensive solutions [38], promoting a sense of ownership and shared cognition within the team that strengthens both teamwork and care outcomes [39]. *Constructive conflict* involves engaging in debates and discussions that challenge assumptions and stimulate critical thinking [40,41]. When disagreements are addressed productively in a culture of psychological safety, conflict becomes a driver of deeper learning, helping teams to refine their approaches and achieve more robust and transformative outcomes. Constructive conflict can also promote a culture of openness and psychological safety [27,38,42].

#### Team-learning mechanisms

According to Decuyper and colleagues [28] two key mechanisms guide team-learning processes over time: team reflexivity and boundary crossing. *Team reflexivity* is an intra-system process in which team members collectively reflect on and adapt their goals, processes, and outcomes. Reflexivity involves deconstructing, co-constructing, and reconstructing collective perceptions and actions of past, present, and future realities [28]. By aligning perceptions and actions dynamically, reflexivity induces a creative tension that drives teams toward learning and improvement in a focused direction [39]. *Boundary crossing*, by contrast, is an inter-system process that establishes or restores continuity in (inter)action across sub-systems, systems, and supra-systems. Within MDTMs, this occurs between professionals from different professions and disciplines, fostering mutual role understanding and the development of interprofessional competence, and between teams and their organizational or supra-systems, helping them integrate diverse standpoints and to adapt to the complexities of integrated care [29]. Together, reflexivity and boundary crossing ensure that MDTMs serve not only as sites of decision-making but also as environments for collective learning and professional growth.

### Constraints of MDTMs as Team-Learning Activity

**Figure 1:**
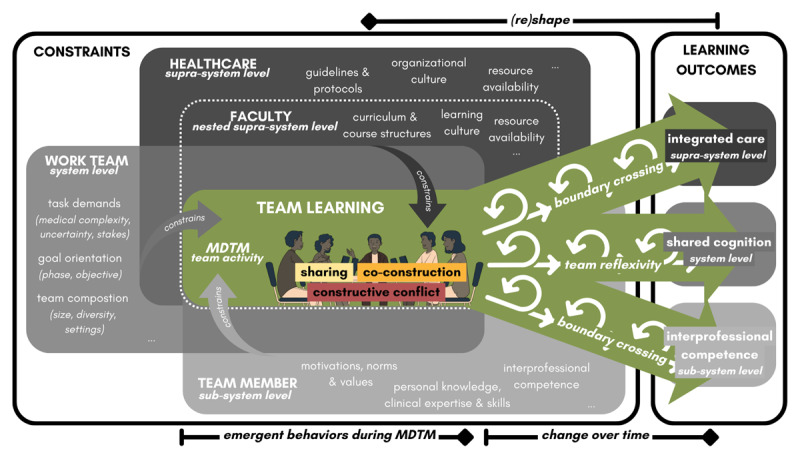
Ecological-dynamical framework for MDTMs as team-learning activity.

Constraints affect how affordances for team learning play out by limiting and shaping the availability of different action possibilities for certain behaviors. Following Decuyper et al. [28] and Mulder [35], we conceptualize work teams as complex open systems with permeable boundaries, interconnecting interdependent sub-systems (team members) both with one another, and with their organizational supra-system (healthcare) and its nested supra-system (medical faculty). Within MDTMs, affordances enable teams to actualize specific team-learning behaviors (e.g., sharing), generating emergent dynamics that foster change over time, such as the development of shared cognition and interprofessional competence. Crucially, engaging in an affordance in a particular way in turn (re)shapes the very constraints that structure the landscape in the moment before, creating iterative cycles of learning and adaptation (see Figure 1).

#### Healthcare/Faculty context

At all of the (nested) supra-systems, affordances for team learning are shaped by contextual constraints [30,36]. In healthcare contexts, constraints arise from guidelines and protocols, organizational culture, and resource availability. In educational contexts, curriculum and course structures, learning culture, and resource availability similarly influence how participation in simulated MDTMs can be constructed as a meaningful team-learning activity.

##### Guidelines and protocols/Curriculum and course structures

Standardized guidelines and protocols enhance consistency by supporting sharing of expertise and structuring decision-making, which improves coordination, reduces variability, and ensures that MDTMs follow established best practices [14]. However, excessive standardization may limit co-construction, as teams have fewer opportunities to adapt creatively to complex, ambiguous or individual-specific needs [43,44]. Conversely, a lack of clear guidelines generates ambiguity that can hinder constructive conflict, as disagreements remain unresolved without shared reference points [43,44]. In faculty contexts, clear educational frameworks and curriculum alignment can similarly invite sharing and co-construction. However, poorly defined structures or rigid curricula may suppress dialogue and critical thinking, limiting MDTMs as authentic team-learning activity [45].

##### Organizational/Learning culture

Culture strongly shapes the availability of different team-learning behaviors. In hierarchical settings, intra- and interprofessional power imbalances may discourage active sharing from members in positions lower in the hierarchy, whose contributions are often judged based on their professional status, formal position, and perceived expertise [42]. Institutions that cultivate inclusivity and openness invite co-construction and constructive conflict, enabling professionals, patients, and families to challenge and integrate standpoints productively [7,43]. Conversely, cultures that undervalue diverse input risk fragmented care planning and reduced learning opportunities [44]. In faculty contexts, open learning cultures encourage students with different backgrounds to engage actively, explore, and learn from mistakes [46]. By contrast, when learning cultures remain siloed, focused narrowly on discipline-specific outcomes, they marginalize the affordances of MDTMs as interprofessional learning environments. Democratizing and establishing a psychologically safe learning environment, where members can contribute openly and challenge ideas constructively, is crucial to sustaining individual and team development [31,42,47].

##### Resource availability

Resources directly constrain affordances for team learning. Adequate infrastructure and support enable sharing (through information systems and meeting spaces), co-construction (by allocating time for dialogue and deliberation), and constructive conflict (by ensuring follow-up and reflection on disagreements). Scarce resources, by contrast, limit the frequency and depth of MDTMs, restricting learning opportunities and decision-making quality [47]. Similarly, in faculty contexts, adequate resources, such as time within the curriculum, simulation facilities, and trained facilitators, are essential for embedding MDTMs as authentic learning activities. Limited faculty investment may result in MDTMs being conducted superficially, without sufficient preparation, debriefing or reflection, thereby undermining their educational value. Conversely, well-supported initiatives for MDTMs with trained facilitators, dedicated time and infrastructure expand the opportunities for all three behaviors, crucial for developing interprofessional competence [46,48].

#### Work team

At the system level, constraints are shaped by task demands, goal orientation, and team composition [28,35]. These constraints shape teams’ capabilities to share knowledge, integrate expertise, and engage in constructive conflict [49,50].

##### Task demands

Demands stem from medical complexity, uncertainty, and stakes [51,52,53]. *Medical complexity* reflects the dynamic interplay between chronic conditions, functional limitations, care utilization, and needs [54,55]. Simple cases might involve isolated health issues with minimal functional impairment, which require little negotiation, while complex cases often involve multimorbidity, significant functional limitations, and substantial support needs, demanding sharing of specialized and personal knowledge and co-construction-integrated solutions. *Uncertainty* reflects the level of ambiguity in patient outcomes, spanning from cases with predictable trajectories to those marked by rare conditions or unpredictable courses [47]. Elevated levels of uncertainty demand continuous information exchange, adaptive problem-solving and openness to alternative perspectives, stimulating all three team-learning behaviors. *Stakes* refer to the potential consequences of decisions, ranging from minor functional risks to high-stakes scenarios involving significant morbidity or mortality, necessitate constructive conflict as teams need to reconcile divergent priorities under pressure [56]. In chaotic, high-stakes and uncertain cases, team members must clarify roles, coordinate rapidly, and iteratively adjust decisions as new information arises [57].

##### Goal orientation

Goal orientation is dynamic and shifts with the *phase* of patient care pathways, from pre-diagnosis to treatment planning and follow-up [58]. The intended outcome of MDTMs -whether diagnostic clarification, treatment decision-making, or long-term management -shapes collaboration and team learning. For instance, diagnostic-focused meetings may emphasize sharing of information; treatment planning often requires co-construction across team members; and long-term care management frequently generates constructive conflict as *objectives* (curative, rehabilitative, or palliative care trajectories) must be re-negotiated in light of evolving needs. The ability to learn and reconfigure in response to changing clinical priorities is critical for effective teamwork and has been identified as a hallmark of high-functioning interprofessional teams [57,59]. Misalignment in goal orientation may impede collaboration, but it also provides opportunities for deeper learning.

##### Team composition

The composition also shapes action possibilities. *Team size* can affect communication and participation: smaller teams often enable more focused co-construction but may limit breadth of expertise, while larger teams provide more opportunities for sharing but risk reduced engagement and coordination [8,47]. *Diversity* across personal, professional, and disciplinary backgrounds of all team members, including patients and their families, enriches problem-solving through sharing and co-construction, and increases the potential for constructive conflicts [60]. Additionally, coordination across primary, secondary, and tertiary care *settings*, each characterized by specific practices and norms, can create opportunities for mutual learning across care boundaries [61]. Effectively structuring team composition, supported by skilled facilitation, can thus turn potential friction into productive learning experiences.

#### Team member

At sub-system level, constraints are shaped by personal knowledge, expertise and skills, motivations, norms and values, and interprofessional competence [28,35]. These constraints define how members perceive and actualize affordances for team learning.

##### Personal knowledge, clinical expertise, and skills

Personal knowledge is partly tacit, embodied in experience and intuition, and held by all team members, including patients and their families. These insights create opportunities for sharing and co-construction. Clinical expertise and skills emerge from the intersection of *profession* (e.g., allied health professionals, physicians, nurses, social workers), *discipline* (e.g., cardiology, geriatrics, psychiatry), and *seniority* (e.g., formal position, perceived expertise), and influences leadership, authority, and participation in discussions. Team members from the same discipline (e.g., radiologists and radiologic technologists) may streamline sharing, while intra-professional teams (e.g., radiologists and general practitioners) engage in co-construction to reconcile divergent approaches [56,60,62]. When essential knowledge or expertise is absent, discussions may lack depth, omit crucial standpoints, and limit decision accuracy and the actualization of learning opportunities. For instance, while a pharmacist ensures medication safety, a radiologist contributes critical imaging insights, or a patient/family member offers contextual knowledge about daily functioning and priorities.

##### Motivations, norms, and values

Individual motivations, norms, and values shape how members act in MDTMs. Intrinsic motivations, such as commitment to care or professional growth, encourage active sharing and engagement in co-construction. Extrinsic motivations, including organizational recognition, team performance and career advancement, may also prompt individual contributions, but sometimes at the cost of team focus [47,63]. When motivation is lacking or primarily self-serving, engagement may be limited, reducing both the quality of collaboration and the actualization of opportunities for co-construction and constructive conflict. Additionally, team members who adhere to hierarchical norms may defer to those with higher perceived authority, limiting sharing and constructive conflict. Patients or families, when guided by similar expectations, may also remain silent even when their experiential insights are essential. By contrast, individuals who value inclusivity, psychological safety, and mutual respect are more likely to share knowledge openly, challenge assumptions constructively, and engage in negotiation [47,64].

##### Interprofessional competence

Interprofessional competence is a key determinant of team effectiveness and learning, and is consistently recognized across international frameworks [10,11,12]. Despite differences in emphasis, these frameworks converge on several domains: *Person-centered* or *relationship-focused care* ensures that patients and families are recognized as contributors of personal knowledge and purposeful relationships between all team members, increasing the opportunities for and engagement in team-learning behaviors [10,11,12]. *Team communication* is geared towards cooperation, responsiveness and respect in teams, focusing on sharing content as well as relational elements [10,11,12]. *Role clarification and negotiation* foster mutual understanding of professional identities, both facilitating and facilitated by opportunities for co-construction [10,11,12]. *Team functioning* emphasizes the importance of understanding the interdependent nature of teams, ensuring time, expertise and contributions are used effectively [10,11,12]. In addition, the Canadian Interprofessional Health Collaboration Competency (CIHC) Framework emphasizes collaborative leadership and the capacity to process team differences and disagreements, both of which stimulate and are stimulated by constructive conflict, a critical driver of deeper learning [10]. Team members who hesitate to share knowledge, avoid co-construction, or lack the skills to engage in constructive conflict can hinder both team learning and decision-making. Conversely, strong interprofessional competence expands the opportunities for all team-learning behaviors, improving integrated care, fostering the development of shared cognition, and further reinforcing interprofessional competence itself.

### MDTMs as Landscape of Affordances

**Figure 2:**
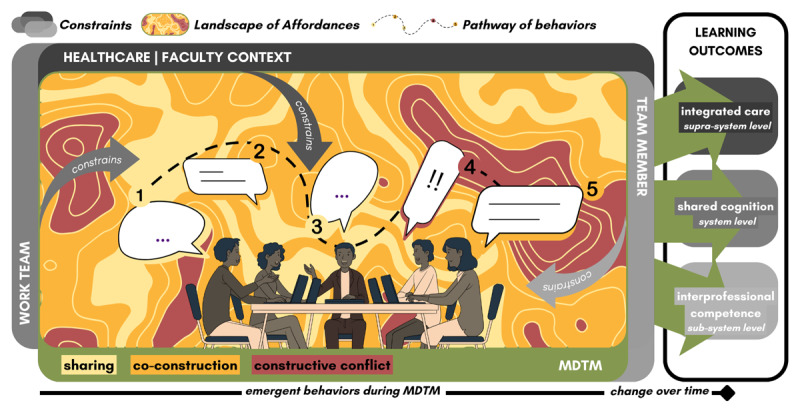
Pathway of behaviors through a Landscape of Affordances constrained by healthcare/faculty context, work team, and team member.

Given the constraints of the healthcare and faculty context, work teams, and individual team members, MDTMs allow for affordances in service of team-learning behaviors, which in turn stimulate processes of individual and team development. The ways of engaging in series of different team-learning behaviors can be conceptualized as a pathway (e.g., sharing → co-construction → sharing → constructive conflict → co-construction) through a Landscape of Affordances, where constraints shape the opportunities for behaviors (e.g., focused areas for constructive conflict, surrounded by wider areas for co-construction and in-between areas for sharing) which can be actualized during the team-learning activity (see Figure 2). Boundary crossing between team members, across teams and contexts supports integrated care by enabling continuity across systems-levels; team reflexivity fosters the development of shared cognition, aligning collective perceptions and actions; and boundary crossing within teams enhances interprofessional competence development by facilitating role clarification, negotiation, and collaborative problem-solving [28,35].

## Constraints-Led Approach to MDTMs

The constraints-led approach, rooted in an ecological-dynamical perspective, emphasizes that learning emerges through exploration and self-organization within constraints [65,66,67]. Rather than prescribing fixed solutions, it focuses on shaping the environment so learners can explore and discover effective responses adaptively. In medical education and beyond, this approach has been shown to improve clinical reasoning, procedural skills, and decision-making by exposing learners to realistic and variable conditions that mirror the complexities of practice [68,69,70,71,72].

**Figure 3:**
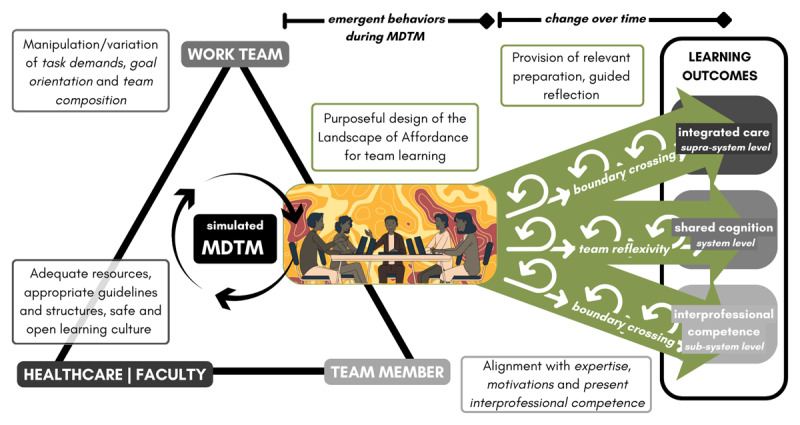
Constraints-led approach to simulated MDTMs as team-learning activity.

Applied to MDTMs as team-learning activity in continuous health profession education, educators can use the constraints-led approach to vary affordances for team learning that facilitate individual and team development by limiting and shaping opportunities for learners to share information, co-construct meaning, and engage in constructive conflict. The degree of control over constraints varies across contexts. For instance, simulated MDTMs offer educators greater scopes to intentionally manipulate constraints, while real-world MDTMs limit direct intervention. Using a backward design approach, they can shape the Landscape of Affordances of simulated MDTMs by varying task demands, goal orientation, and team composition to promote shared cognition at the team level and interprofessional competence at the individual level [73,74] (see Figure 3). For instance, a highly complex case (e.g., care planning for children with medical complexity) may invite wider discussions among learners with appropriate levels of expertise and competency, thereby promoting deep learning. Conversely, the absence of a key learner (e.g., a radiologist in a case requiring detailed imaging review) may restrict meaningful decision-making and learning, illustrating how constraints shape team interactions. Effective simulation scenarios intentionally reflect the demands of clinical practice while ensuring optimal learning trajectories. Matching learners’ expertise to case complexity enhances engagement, while structured dialogue, guided supervision, and reflective debriefing reinforce key team-learning behaviors and facilitate learning outcomes across system levels [13,75]. More specifically, to strengthen role identification and negotiation, learners must have opportunities to share discipline-specific insights and access essential guidelines, protocols, and decision-supporting tools. Simulated MDTMs should integrate clinical content and interprofessional competency development, ensuring that collaboration is not merely an adjunct to clinical decision-making but an essential element of the learning process [65]. Collaborative leadership is promoted by structuring scenarios in which each learner holds distinct but interdependent information, making contributions essential to team problem-solving [76]. Constructive conflict, a key driver of deeper learning, is encouraged through the deliberate introduction of conflicting perspectives, ambiguous information, and diverse team compositions, compelling learners to negotiate solutions collaboratively [28,42]. A process-oriented approach that integrates repeated cycles of relevant preparation, collaborative engagement, and guided reflection further reinforces the development of shared cognition and interprofessional competence over time [28].

In a clinical context, where learners observe or participate in real-world MDTMs, the role of educators is primarily one of selection: choosing MDTMs that provide particular information about affordances for learners, preparing them with relevant background materials, and facilitating structured reflection afterwards. Such scaffolding helps learners observe or participate in ways that stimulate the processes of team reflexivity and boundary crossing, supporting the development of shared cognition and interprofessional competence [28]. By contrast, simply allowing learners to attend MDTMs without clear learning goals risks superficial engagement and limited educational value.

Finally, the constraints-led approach recognizes that team and individual development in MDTMs is inherently non-linear, progressing through cycles of stability and instability as shared cognition and interprofessional competence emerge and refine [65,68]. Educators should therefore continuously adjust constraints over time, modulating task demands, team composition, and goal orientation, to align with intended learning outcomes at organizational, team, and individual levels. By incorporating scenarios with varying complexity, uncertainty, and stakes, they can create adaptive learning environments that actualize the transformative potential of MDTMs as team-learning activity, preparing learners for the dynamic and unpredictable realities of integrated care.

## Conclusion

This paper introduced a conceptual framework grounded in an ecological-dynamical perspective to systematically understand team learning in MDTMs. Within this framework, sharing, co-construction, and constructive conflict are enacted as team-learning behaviors, while reflexivity and boundary crossing serve as processes that foster interprofessional competence, shared cognition, and integrated care. By examining constraints across 1) healthcare and faculty contexts, 2) work teams, and 3) team members, we showed how these conditions shape the Landscape of Affordances from which specific learning behaviors can be actualized. Building on this foundation, the constraints-led approach provides practical guidance for deliberately selecting, designing, and varying constraints to transform simulated MDTMs into authentic and adaptive learning environments. This approach enhances both education and practice: enabling learners to develop interprofessional competence for the complexities of integrated care, while ensuring MDTMs evolve from coordination-focused discussions toward more flexible and reflexive models of collaboration through shared cognition. Taken together, the ecological-dynamical framework offers a systematic understanding of MDTMs as team-learning activity, while the constraints-led approach harnesses their transformative potential to strengthen adaptive team performance, interprofessional development, and the delivery of integrated care in complex healthcare systems. More broadly, the conceptual framework may provide a foundation for future research, while the constraints-led approach offers a guide that can be further investigated and empirically grounded [77].
